# Effect of Vitamin K Supplementation on Testosterone Production in a Rat Model of Late-Onset Hypogonadism

**DOI:** 10.3390/foods15061070

**Published:** 2026-03-18

**Authors:** Rui Murakami, Yusuke Ohsaki, Hikaru Ito, Hsin-Jung Ho, Afifah Zahra Agista, Yi-Fen Chiang, Ya-Ling Chen, Masamitsu Maekawa, Takuo Hirose, Kenshiro Hara, Wan-Chun Chiu, Chiu-Li Yeh, Shih-Min Hsia, Suh-Ching Yang, Nariyasu Mano, Takefumi Mori, Hitoshi Shirakawa

**Affiliations:** 1 Laboratory of Nutrition, Graduate School of Agricultural Science, Tohoku University, Sendai 980-8572, Japanhjho@tohoku.ac.jp (H.-J.H.); agista@g-mail.tohoku-university.jp (A.Z.A.); shirakah@tohoku.ac.jp (H.S.); 2 International Education and Research Center for Food Agricultural Immunology, Graduate School of Agricultural Science, Tohoku University, Sendai 980-8572, Japan; 3 Institute for Excellence in Higher Education, Tohoku University, Sendai 980-8576, Japan; 4 School of Nutrition and Health Sciences, College of Nutrition, Taipei Medical University, Taipei 11031, Taiwan; yifenchiang@tmu.edu.tw (Y.-F.C.); ylchen01@tmu.edu.tw (Y.-L.C.); wanchun@tmu.edu.tw (W.-C.C.); clyeh@tmu.edu.tw (C.-L.Y.); bryanhsia@tmu.edu.tw (S.-M.H.); sokei@tmu.edu.tw (S.-C.Y.); 5 Department of Pharmaceutical Sciences, Tohoku University Hospital, Sendai 980-8574, Japan; masamitsu.maekawa.a2@tohoku.ac.jp (M.M.); nariyasu.mano.c8@tohoku.ac.jp (N.M.); 6 Division of Nephrology and Hypertension, Faculty of Medicine, Tohoku Medical and Pharmaceutical University, Sendai 983-8536, Japan; hirose.takuo@tohoku-mpu.ac.jp (T.H.); tmori@tohoku-mpu.ac.jp (T.M.); 7 Division of Integrative Renal Replacement Therapy, Faculty of Medicine, Tohoku Medical and Pharmaceutical University, Sendai 983-8536, Japan; 8 Laboratory of Animal Reproduction and Development, Graduate School of Agricultural Science, Tohoku University, Sendai 980-8572, Japan; kenshiro.hara.b6@tohoku.ac.jp

**Keywords:** vitamin K, menaquinone-4, late-onset hypogonadism, testosterone

## Abstract

Late-onset hypogonadism (LOH) is an age-related condition characterized by a decline in testosterone (Ts) levels and associated symptoms that impair quality of life in older men. Although Ts replacement therapy is available, its clinical use is limited by adverse effects. Vitamin K (VK) is a fat-soluble vitamin that functions as a cofactor for γ-glutamylcarboxylase and plays important roles in blood coagulation and bone homeostasis. Menaquinone-4 (MK-4), a VK homolog predominantly found in animal-derived foods, has been shown to enhance Ts production in healthy male rats. However, whether this effect occurs under low-Ts conditions remains unclear. In this study, we investigated the effects of VK on LOH using a leuprorelin acetate (LA)-induced low-Ts rat model. Male Sprague–Dawley rats were administered sustained-release LA and fed a control diet or diets supplemented with VK1 or MK-4 (75 mg/kg) for 4 weeks. Compared with the control group, MK-4 supplementation significantly ameliorated the reduction in serum Ts levels and seminiferous tubule diameter, whereas VK1 supplementation showed no significant effects. Furthermore, MK-4 supplementation activated the protein kinase A signaling pathway, which is directly involved in testicular Ts production. These findings suggest that MK-4 supplementation may represent a novel nutritional strategy for the management of LOH.

## 1. Introduction

According to the United Nations World Population Prospects 2024, population ageing is accelerating worldwide, and the number of people aged 65 years and older is projected to reach approximately 2.2 billion by the late 2070s [1]. With such changes, maintaining the quality of life and extending the healthy life expectancy of older adults have become global goals. With advancing age, the capacity of multiple organs to maintain physiological homeostasis progressively declines, increasing the risk of several types of cancer and lifestyle-related diseases [2,3]. The testes play a crucial role in maintaining the reproductive system, muscle, brain, and bone functions by producing testosterone (Ts), the major male sex hormone [4]. Leydig cells, which produce Ts in the testes, are susceptible to aging [5]; blood Ts levels peak in the 20s–30s and then decline with age [6]. A reduction in Ts production leads to the development of late-onset hypogonadism (LOH), which is characterized by decreased blood Ts levels because of aging, accompanied by various symptoms, including sexual dysfunction, muscle weakness, and increased body fat mass, and may even cause psychological disorders, such as depression and loss of libido [7]. In addition, previous studies have suggested that long-term Ts deficiency increases the risk of all-cause mortality [8], diabetes [9], and cardiovascular diseases [10]. Ts replacement therapy (TRT) is available to treat LOH [11]; however, TRT has been reported to have adverse effects that may worsen prostate disease, exacerbate sleep apnea, and impair spermatogenic function, thus limiting its clinical use [12,13]. Considering the rapid progression toward a super-aging society, novel therapeutic approaches for LOH are an urgent priority.

Vitamin K is a fat-soluble vitamin that exists in two forms in food: vitamin K1 (VK1, phylloquinone), which is abundant in green vegetables, and vitamin K2 (VK2, menaquinone), which is primarily found in fermented foods produced by microorganisms and animal products. Vitamin K is involved in the blood coagulation system by acting as a cofactor responsible for the post-translational modification of glutamate residues into γ-carboxy glutamate residues in certain proteins [14]. Some studies have revealed other physiological functions, including the regulation of bone homeostasis [15,16], anti-inflammation [17], anti-ferroptosis [18], and amelioration of mitochondrial damage [19]. We previously revealed that menaquinone-4 (MK-4), the predominant homolog of vitamin K2 found at high concentrations in rat testes [20], plays an essential role in the production or secretion of male sex hormones [21]. Feeding male rats a vitamin K-deficient diet can cause low-Ts levels in the testes and serum [21]. Furthermore, MK-4 supplementation increased Ts production in the testes of healthy male rats [22] and in LPS-induced inflammatory male rats [23]. However, studies using LOH animal models are required because of the uncertainty regarding whether the same effects can be observed under low-Ts symptoms.

Naturally aged [5,24,25] or diet-induced obese animals [26,27] are commonly used as LOH model animals. However, these methods require considerable time to induce LOH-like symptoms, and some studies have reported that these methods do not reduce blood Ts levels [28,29]. A sustained-release gonadotropin-releasing hormone (GnRH) agonist, leuprorelin acetate (LA), which continuously decreases Ts production in the testes by downregulating GnRH receptors, has been used to treat human prostate cancer and diseases caused by excessive Ts production [30,31]. Serum Ts levels decrease in non-aged male rats receiving LA injection, allowing the preparation of LOH model rats [32,33]. Hence, this study was conducted to clarify the effects of vitamin K supplementation on low-Ts symptoms in LA-induced LOH model rats.

## 2. Materials and Methods

### 2.1. Materials

MK-4 (purity: >98.0%) was purchased from Tokyo Chemical Industry (Tokyo, Japan). VK1 (purity: >97.0%) and the other reagents were obtained from FUJIFILM Wako Pure Chemical Co. (Osaka, Japan). Other reagents used in this study were of analytical grade.

### 2.2. Animals and Treatments

Seven-week-old male Sprague–Dawley (SD) rats were purchased from SLC Japan (Shizuoka, Japan) and housed in the animal facility of Tohoku University. Each rat was housed in a cage at 23 ± 2 °C under a 12/12 h light-dark cycle (lights on at 8:00 a.m.). All protocols were approved by the Animal Research and Animal Care Committee, Tohoku University (Approval number: 2021AgA-003).

**Figure 1:**
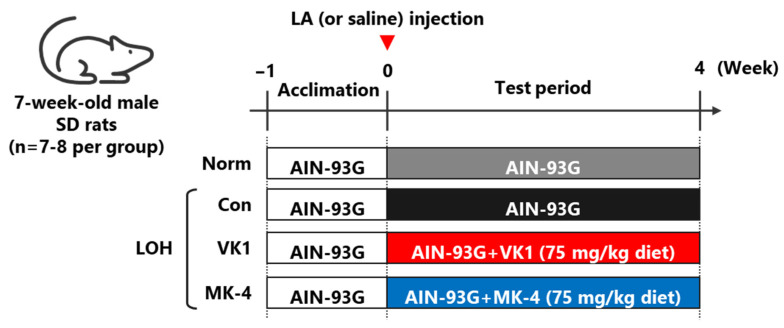
Schematic representation of the experimental workflow.

Rats were randomly separated into four groups: (a) normal group (Norm, *n* = 7): rats fed the AIN-93G diet (Oriental Yeast Co., Tokyo, Japan); (b) LOH control group (Con, *n* = 8): rats fed the AIN-93G diet; (c) LOH+VK1 group (VK1, *n* = 8): rats fed the AIN-93G diet containing 75 mg/kg VK1; and (d) LOH+MK-4 group (MK-4, *n* = 7): AIN-93G diet containing 75 mg/kg MK-4. The dosage of vitamin K was set the same as our previous study [22]. To prepare LOH model rats, LA formula (Nipro Co., Osaka, Japan) at a dose of 1.5 mg/kg body weight was injected subcutaneously into the rats at the beginning of the experiment, whereas normal rats received the same amount of phosphate-buffered saline (PBS). The rats were fed diets with or without VK1 or MK-4 for 4 weeks and were subsequently euthanized. The testes were collected, frozen in liquid nitrogen, and stored at −80 °C until further use. During the experimental period, the rats were allowed full access to their diet and drinking water. Blood was collected from the tail vein one day before the end of the experimental period to obtain serum (Figure 1).

### 2.3. Measurement of Vitamin K Content in Rats’ Testes

Testicular vitamin K was extracted as described previously [34]. Briefly, the testes (approximately 0.1 g) were homogenized in 2 mL of 2-propanol (66% water), followed by the addition of 5 mL *n*-hexane. Subsequently, 1 mL of an internal standard (menaquinone-3, Eisai Co., Tokyo, Japan; 9.92 ng/mL) was added. The samples were centrifuged at 1600× *g* for 5 min, and the upper layers were collected and evaporated. After resuspension in 2 mL *n*-hexane, the samples were fractionated using Sep-Pak Plus Silica (Waters, Milford, MA, USA). The eluted fractions (4% diethyl ether in *n*-hexane) were evaporated, resuspended with 200 µL of ethanol, filtered using DISMIC-03JP (ADVANTEC, Kanagawa, Japan), and measured by fluorescent high-performance liquid chromatography (HPLC) using an Agilent 1260 Infinity system (Puresil 5C_18_ column, Waters; RC 10-3 PtO_2_ column, Shiseido-Irica, Kyoto, Japan; excitation at 240 nm, emission at 430 nm). The VK1 and MK-4 concentrations in the samples were determined by measuring the fluorescence intensity relative to the internal standard.

### 2.4. Measurement of Ts Concentrations in the Serum and Testes of Rats

Testes (0.1 g) were homogenized in 500 µL PBS, and the homogenates were centrifuged at 14,000× *g* for 3 min at 4 °C. Ts in the supernatant were extracted using five volumes of diethyl ether. The extracts were centrifuged at 900× *g* for 5 min at 4 °C, and the ether layer was collected. This process was repeated thrice, and the collected ether was evaporated using a vacuum-centrifugal evaporator. The extracts were resuspended in ELISA Buffer (Cayman Chemical, Ann Arbor, MI, USA). Serum Ts was extracted and collected as described previously. Ts measurements were conducted using liquid chromatography-tandem mass spectrometry (LC-MS/MS), as previously described [35]. Briefly, 10 µL of the samples were mixed with 100 µL of deuterium-labeled Ts (300 pg/mL) as an internal standard solution, evaporated under N_2_ gas, and resuspended with 50% methanol. A QTRAP 6500 linear ion trap-quadrupole hybrid tandem mass spectrometer (SCIEX, Framingham, MA, USA) connected to a Nexera series ultra-high-performance liquid chromatograph (Shimadzu, Kyoto, Japan) and equipped with a CAPCELL CORE C_18_ column (2.1 mm inner diameter × 150 mm, 2.7 µm, 40 °C; Osaka Soda; Osaka, Japan) was used for the measurements. For the separation of Ts in the samples, mobile phase A comprised formic acid/water (0.1:100, *v*/*v*) and mobile phase B comprised formic acid/methanol/acetonitrile (0.1:50:50, *v*/*v*/*v*). The mobile phase was delivered using a linear gradient at a flow rate of 0.4 mL/min. Data were analyzed using Analyst 1.6.2 and MultiQuant software 2.1.1 (SCIEX).

### 2.5. Histological Analysis

Testicular tissue was fixed in 10% formalin solution, dehydrated with ethanol at various concentrations, and embedded in paraffin. Tissue sections were prepared, hematoxylin and eosin (HE) staining was performed, and image data acquisition was outsourced to the Histopathology Core Facility at Tohoku Medical and Pharmaceutical University. Regions of interest (ROIs) were randomly selected for each sample, and the diameters of all seminiferous tubules in the ROI were measured using the ImageJ software (version 1.54r, NIH, Bethesda, MD, USA). Subsequently, seminiferous tubules with circularity less than 0.8 were excluded to eliminate the longitudinally sectioned tubules [36]. Subsequently, 40 tubules from each sample were randomly selected for analysis.

### 2.6. Measurement of mRNA Expression

Testis samples preserved at −80 °C were used for quantitative reverse transcriptase-mediated polymerase chain reaction (RT-qPCR), following a previously described procedure [37]. Briefly, total RNA was extracted using ISOGEN reagent (Nippon Gene Co., Ltd., Tokyo, Japan) according to the manufacturer’s instructions. The quality and concentration of the extracted RNA were determined by measuring the absorbance at 260 and 280 nm using a spectrophotometer. The resulting RNA was denatured by incubation with 50 ng/μL oligo-dT primers (Hokkaido System Science Co., Sapporo, Japan) and 1 mM dNTP (GE Healthcare, Tokyo, Japan) at 65 °C for 5 min. Denatured RNA was used as a template to synthesize cDNA by mixing it with an RT buffer (50 mM Tris-HCl at pH 8.3, 75 mM KCl, 3 mM MgCl_2_, and 5 mM dithiothreitol) containing 50 U SuperScript III reverse transcriptase (Invitrogen, Carlsbad, CA, USA) and 20 U RNaseOUT RNase inhibitor (Invitrogen), followed by incubation at 50 °C for 60 min. The synthesized cDNA was used to amplify the target sequences using gene-specific primers (Table 1) and TB Green Premix Ex Taq (Takara Bio, Otsu, Japan) by qPCR in a CFX Connect Real-Time PCR Detection System (Bio-Rad Laboratories, Inc., Hercules, CA, USA). The results were normalized to those of the eukaryotic elongation factor 1α1 (*Eef1a1*).

### 2.7. Measurement of Protein Expression

Rat testes (0.1 g) were homogenized in 500 µL of PBS containing protease inhibitors (Complete mini protease inhibitor cocktail, Merck, Darmstadt, Germany) and phosphatase inhibitors (PhosSTOP EASYpack, Merck). Samples were centrifuged at 14,000× *g* and 4 °C for 5 min. Protein concentrations in the collected supernatants were determined using a Bio-Rad Protein Assay (Bio-Rad Laboratories). Subsequently, the samples were diluted, denatured in SDS gel loading buffer, resolved by 12.5% SDS-polyacrylamide gel electrophoresis using SuperSep Ace (FUJIFILM Wako Pure Chemical), and transferred onto polyvinylidene fluoride membranes (Millipore, Burlington, MA, USA). After blocking with Tris-buffered saline Tween-20 (TBS-T) containing 3% bovine serum albumin (BSA) for 1 h, the membrane was incubated in 3% BSA-TBS-T containing a primary antibody overnight at 4 °C. The membrane was washed thrice with 3% BSA-TBS-T for 8 min, followed by incubation in 3% BSA-TBS-T containing a secondary antibody for 1 h at room temperature. Finally, the membrane was washed as previously described, and signals were detected using Immobilon Western Chemiluminescent HRP Substrate (Millipore) and ChemiDoc Touch Imaging System (Bio-Rad Laboratories).

The antibodies used were anti-protein kinase A (PKA, cat. no. 4782; Cell Signaling Technology, Danvers, MA, USA; 1:1000) and anti-phospho PKA (cat. no. 4781; Cell Signaling; 1:1000) and anti-α-tubulin (cat. No. T5168; Sigma-Aldrich, St. Louis, MO, USA;; 1:10,000) as the primary antibody and anti-rabbit IgG (cat. no. 31460; Thermo Fisher Scientific, Waltham, MA, USA; 1:5000) and anti-mouse IgG (cat. no. 32230; Thermo Fisher Scientific, 1:10,000) were used as secondary antibodies.

### 2.8. Statistical Analysis

All data are expressed as mean ± standard error (SEM). Statistical analyses were performed using StatcelQC (OMS Publishing, Saitama, Japan) and SigmaPlot 12 (Systat Software Inc., Chicago, IL, USA). For all data, a two-group comparison was performed between the Norm and Con groups to confirm that the LOH model animals were successfully generated by the LA injection. The three LA-injected groups were compared to assess the effects of vitamin K supplementation. For body weight and food intake, a two-way repeated-measures analysis of variance (two-way RM ANOVA) followed by Dunnett’s post hoc test was performed. For other data, Student’s *t*-test was performed to compare the Norm and Con groups, and one-way ANOVA followed by Dunnett’s post hoc test was conducted to compare the LA-injected groups. Differences were considered statistically significant at *p* < 0.05.

## 3. Results

### 3.1. Effects of LA and Vitamin K Administration on Body Weight and Testicular Weight in Rats

**Figure 2:**
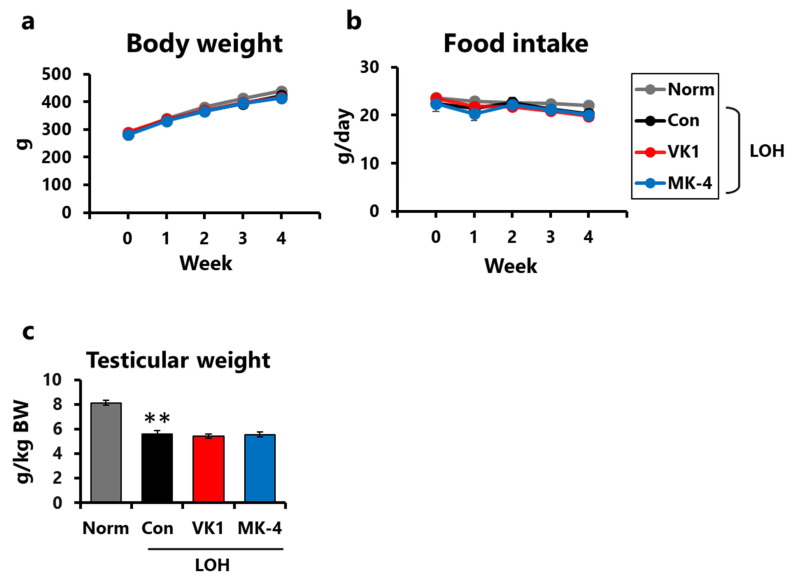
Vitamin K supplementation did not affect body weight, food intake, or testicular weight. (**a**) Body weights. (**b**) Food intake. (**c**) Testicular weight adjusted for body weight. BW, body weight. Data are presented as mean ± SEM (n = 7–8). Two-way RM ANOVA, Dunnett’s test (vs. Norm or Con among LOH groups). (**a**,**b**). Student’s *t*-test (Norm vs. Con). One-way ANOVA, Dunnett (vs. Con among the LOH groups) (**c**). ** *p* < 0.01 (vs. Norm).

No significant differences were observed in body weight or food intake among the groups during the study period (Figure 2a,b). Relative testicular weight (testicular weight/body weight) was significantly decreased by LA injection (LOH groups), suggesting that LA injection induced testicular atrophy (Figure 2c). However, vitamin K supplementation did not mitigate these effects.

### 3.2. Accumulation of Dietary Vitamin K in Rats’ Testes

**Figure 3:**
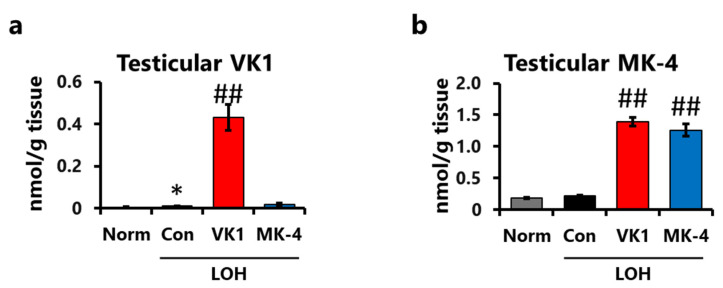
Dietary vitamin K accumulated in the testes, predominantly as MK-4. (**a**) Testicular VK1 and (**b**) MK-4 levels. Data are presented as mean ± SEM (*n* = 7–8). Student’s *t*-test (Norm vs. Con). One-way ANOVA, Dunnett (vs. Con) among the LOH groups. * *p* < 0.05 (vs. Norm), ## *p* < 0.01 (vs. Con).

To assess the changes in vitamin K levels in the testes, testicular VK1 and MK-4 levels were determined using a fluorescent HPLC system. Testicular VK1 levels were higher in the VK1 group than those in the normal group (Figure 3a), whereas testicular MK-4 levels in both the VK1 and MK-4 groups were significantly higher and comparable (Figure 3b). These results indicate that dietary VK1 and MK-4 accumulate in the testes and that part of the dietary VK1 is converted into MK-4 after ingestion.

### 3.3. Effects of LA and Vitamin K Administration on Ts Production and Testicular Inner Structure

**Figure 4:**
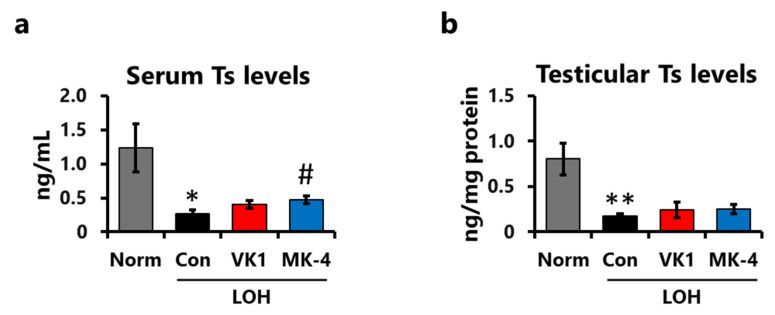
MK-4 supplementation ameliorated the decrease in serum Ts levels induced by LA injection at week 4. (**a**) Serum Ts levels at week 4. (**b**) Testicular Ts levels adjusted by the protein concentration of testis homogenates. Data are presented as means ± SEM (*n* = 7–8). Student’s *t*-test (Norm vs. Con). One-way ANOVA, Dunnett (vs. Con) among the LOH groups. * *p* < 0.05, ** *p* < 0.01 (vs. Norm), # *p* < 0.05 (vs. Con).

The Ts levels in the serum and testes were measured using LC-MS/MS. Compared to the Norm group, serum Ts levels were significantly decreased 4 weeks after LA injection (Figure 4a). Serum Ts levels in the MK-4 group were significantly higher than those in the control group (Figure 4a). Meanwhile, the levels in the VK1 group were slightly higher, but the difference was not statistically significant. Consistent with serum Ts levels, testicular Ts levels also decreased following LA injection; however, neither VK1 nor MK-4 supplementation alleviated this reduction (Figure 4b). This indicates that MK-4 supplementation, but not VK1 supplementation, ameliorated the reduction in serum Ts levels caused by LA treatment.

**Figure 5:**
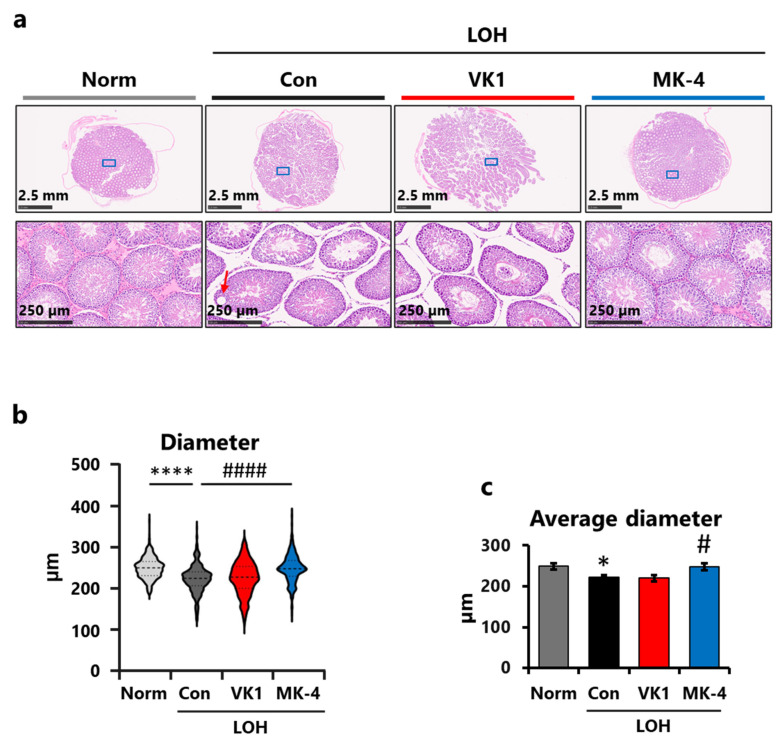
MK-4 supplementation alleviated LA-induced atrophy of seminiferous tubules. (**a**) Representative images of testicular sections from each group. Blue boxes indicate the locations of the enlarged images of each sample, whereas the red arrow indicates the area of epithelial vacuolation. (**b**) Distribution of seminiferous tubule diameter. (**c**) Average seminiferous tubule diameter. Data are presented using violin plots (*n* = 280–320) or as means ± SEM (*n* = 7–8). Student’s *t*-test (Norm vs. Con). One-way ANOVA, Dunnett (vs. Con) among the LOH groups. * *p* < 0.05, **** *p* < 0.001 (vs. Norm), # *p* < 0.05, #### *p* < 0.001 (vs. Con).

HE-stained testicular specimens were used to assess the effects of LA and vitamin K administration on testicular morphology. Compared to the Norm group, loss of germ cells, influx of immature germ cells into the seminiferous lumen, and vacuolated epithelium (red arrow) were observed in the control group (Figure 5a). ImageJ-based quantitative analysis also revealed that LA injection resulted in a downward shift in the distribution of seminiferous tubules (Figure 5b), whereas an upward shift was observed in the MK-4 group compared with the Con group. Similarly, the average diameters were significantly shorter in the Con group than in the Norm group and improved in the MK-4 group compared to the Con group. These findings suggest that MK-4 supplementation can mitigate seminiferous tubule atrophy by enhancing Ts production.

### 3.4. Effect of Vitamin K on the Expression Levels of Factors Associated with Ts Production

**Figure 6:**
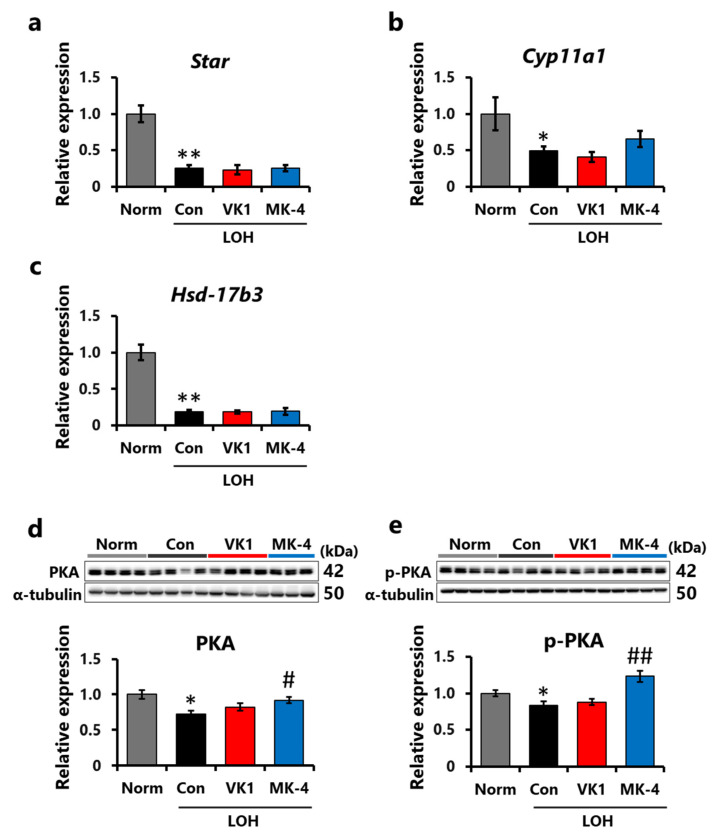
MK-4 supplementation reversed the suppression of the PKA pathway in the testes induced by LA injection. (**a**–**c**) mRNA expression levels of steroidogenic genes in the testes. The expression levels were normalized to those of *Eef1a1*. (**d**) PKA and (**e**) p-PKA protein expression levels in the testes. The expression levels were normalized to those of α-tubulin. Data are presented as means ± SEM (*n* = 7–8). Student’s *t*-test (Norm vs. Con). One-way ANOVA, Dunnett (vs. Con) among the LOH groups. * *p* < 0.05, ** *p* < 0.01 (vs. Norm), # *p* < 0.05, ## *p* < 0.01 (vs. Con).

To investigate the mechanism of action of MK-4 on Ts production in LA-induced LOH rats, RT-qPCR was used to analyze the expression levels of *Star*, which is involved in the transport of cholesterol into the mitochondria; *Cyp11a1*, the rate-limiting enzyme for Ts production; and *Hsd17b3*, which catalyzes the final step of Ts production [38]. Compared to the Norm group, the mRNA expression of *Star*, *Cyp11a1*, and *Hsd17b3* decreased in the Con group (Figure 6a–c). These results indicate that the expression levels of these three genes were downregulated by LA injection. Compared to the Con group, *Cyp11a1* expression levels in the MK-4 group increased (Figure 6b), whereas the expression of *Star* and *Hsd17b3* was not altered by MK-4 supplementation (Figure 6a,c).

In addition, the interaction between pituitary luteinizing hormone (LH) and its corresponding receptor increases cAMP levels in Leydig cells via adenyl cyclase activation, followed by PKA phosphorylation, which leads to Ts synthesis in the testes [39]. Hence, the protein expression of testicular PKA and p-PKA was assessed using Western blotting. The results showed a reduction in both PKA and p-PKA levels following LA injection, whereas MK-4 supplementation significantly increased these levels (Figure 6d,e). These results indicate that the enhancement of Ts production by MK-4 may be attributed to the activation of the cAMP/PKA pathway.

## 4. Discussion

Ts is primarily produced in the testes under the control of the hypothalamic-pituitary-testicular axis [40]. Testicular atrophy and low-Ts levels are typical characteristics of aging testes [41]. A previous study reported that the sensitivity or expression levels of GnRH receptors in the pituitary gland can decrease with age, leading to low-Ts levels [42]. In the current study, we established LOH model animals by injecting them with an excess dose of LA, a sustained-release GnRH agonist. As Ts play a major role in the survival and differentiation of testicular germ cells [43], a single subcutaneous injection of LA caused testicular atrophy with a reduction in testicular weight, accompanied by decreased serum and testicular Ts levels, and atrophy of seminiferous tubules in the current study. Therefore, the LA injection model is useful for investigating Ts deficiency and LOH.

In this study, we compared the effects of dietary supplementation with VK1 and MK-4 on LOH model. VK1 and MK-4 have the same naphthoquinone structure but different side-chain structures. Dietary vitamin K is partially converted into MK-4 within the tissues after absorption in the small intestine [20]. Consistent with this, a high concentration of MK-4 accumulated in the testes of both the MK-4 and VK1 groups. The recovery of serum and testicular Ts levels and seminiferous tubule diameter observed in the MK-4 group was not seen in the VK1 group. We previously found that treatment with MK-4, but not VK1, enhanced Ts production in mouse testis-derived I-10 cells [22], suggesting that VK1 and MK-4 may play different roles in Ts production and testicular functions. However, the unexpected effects of increased testicular VK1 and MK-4 levels in the VK1 group are unclear. One explanation is the competitive interaction between VK1 and MK-4 in the regulation of Ts within the testes; however, further studies are required to clarify this mechanism.

When GnRH is released from the hypothalamus and binds to its receptors in the pituitary gland, it is secreted into the bloodstream and binds to LH receptors on Leydig cells in the testes [39]. Subsequently, adenylyl cyclase is activated, accompanied by the upregulation of downstream factors, including PKA, cAMP response element-binding protein, steroidogenesis acute regulatory protein (StAR), and other steroidogenesis-related enzymes such as CYP11A1, which leads to Ts synthesis. We also found a decrease in the mRNA expression of *Star*, *Cyp11a1*, and *Hsd17b3* in the LOH model rats. In contrast, we previously found that the effects of MK-4 on Ts production were abolished by a PKA inhibitor, H89, but not by warfarin, which inhibits vitamin K epoxide reductase and leads to the suppression of γ-carboxylation in I-10 cells [22]. Moreover, geranylgeraniol, a side-chain structural analog of MK-4, enhances Ts production in I-10 cells [44]. These results suggest that the effects of MK-4 on Ts production are not mediated by the γ-carboxylation of a vitamin K-dependent protein but through the activation of the PKA-dependent pathway. In this study, the recovery effect of MK-4 supplementation on reduced Ts production was thought to involve the activation of the PKA-dependent pathway, similar to the findings of previous studies. In addition, the side-chain structure of MK-4 may enhance Ts production in rats with LA-induced LOH. Verifying this hypothesis requires further research, including elucidation of the detailed molecular mechanism by which MK-4 and its side-chain structure activate PKA.

In a previous study using LH receptor-deficient mice, the lack of LH signaling resulted in a shortened diameter of the seminiferous tubules, and TRT reversed this morphological change [45]. The ratio of the risks to benefits of TRT is unclear; thus, it has been proposed that TRT should be used in aged male populations with remarkably low-Ts levels and obvious symptoms of hypogonadism [46,47]. Several studies have been conducted on the effects of food components, herbal medicines [48], and their active ingredients [49,50] on the improvement of low-Ts levels; however, few components have established safety in humans following long-term or high-dose administration. In contrast, vitamin K is a safe dietary component, and adverse effects associated with its excessive intake have not been reported at clinically relevant doses. Furthermore, when the MK-4 dose used in the current study was converted to a human-equivalent dose, it was approximately 38 mg/day [51], which is lower than the dose currently used for the treatment of osteoporosis in Japan (45 mg/day) [52]. Therefore, MK-4 administration is expected to become a useful and safer alternative therapeutic option for LOH, particularly in cases of borderline androgen deficiency, in which TRT is avoided.

Several previous studies showed that the expression levels of inflammatory factors such as IL-1β, IL-6, TNF-α, or COX-2 are elevated because of aging, which leads to decreased Ts production [53,54], and anti-inflammatory drugs, including a COX-2 inhibitor, have the potential to suppress the reduction in Ts production [55]. In our previous study, we revealed that MK-4 can suppress lipopolysaccharide (LPS)-induced inflammatory reactions by regulating NF-κB activity [17,34]. In addition, we have found that dietary vitamin K alleviates the reduction in Ts production and the elevation of *Nf-κb* mRNA expression induced by LPS administration [23]. However, in the testes of the LOH model rats used in this study, increased expression levels of inflammatory cytokines such as *Il-1β*, *Il-6*, or *Tnf-α* were not observed. Therefore, the effect of MK-4 on Ts production observed in this study was not mediated through an inflammatory pathway.

## 5. Conclusions

To our knowledge, this study is the first to demonstrate that dietary administration of MK-4, a form of vitamin K, can enhance Ts production via activation of the PKA-dependent pathway, even under conditions of low-Ts symptoms in vivo. This suggests that MK-4 supplementation may be a safe and promising alternative therapeutic strategy for treating LOH.

Despite these findings, this study has several limitations. First, the LA-injected model does not adequately replicate the complex physiological conditions associated with aging. This model focuses only on the cAMP/PKA pathway in the testes and is not appropriate for assessing the effects of MK-4 on oxidative stress or inflammation, which are commonly observed in aged testes [56]. Moreover, aging has been reported to alter intestinal vitamin K absorption [57], which may influence the beneficial effects of dietary MK-4 on LOH. Second, the effects of MK-4 on tissues other than the testes, as well as on reproductive capacity, were not evaluated. Ts exerts diverse functions in multiple tissues beyond the testes, including the brain and skeletal muscles, and assessment of its effects in these tissues would require long-term experimental studies.

## Figures and Tables

**Table 1:** List of nucleotide sequences of gene-specific primers.

Gene Symbol	Forward Primer (5′→3′)	Reverse Primer (5′→3′)
*Eef1a1*	GATGGCCCCAAATTCTTGAAG	GGACCATGTCAACAATTGCAG
*Cyp11a1*	GGCCCCATTTACAGGGAGAAG	CTCGCAGGAGAAGAGTGTCG
*Hsd17b3*	TCTGCAAGGCTTTACCAGGG	ATGTCTGGCCAGCTCAAATG
*Star*	CAGTATTGACCTCAAGGGGTGG	TGGCTGGCGAACTCTATCTG

## Data Availability

The original contributions presented in this study are included in the article. Further inquiries can be directed to the corresponding author.

## Abbreviations

The following abbreviations are used in this manuscript:

GnRH	Gonadotropin-releasing hormone
LA	Leuprorelin acetate
LOH	Late-onset hypogonadism
MK-4	Menaquinone-4
PKA	Protein kinase A
TRT	Testosterone replacement therapy
Ts	Testosterone
VK1	Vitamin K1
